# Rapid brain death caused by a cerebellar abscess with *Fusobacterium nucleatum* in a young man with drug abuse: a case report

**DOI:** 10.1186/1756-0500-7-353

**Published:** 2014-06-10

**Authors:** Gunnar TR Hischebeth, Vera C Keil, Katrin Gentil, Azize Boström, Klaus Kuchelmeister, Isabelle Bekeredjian-Ding

**Affiliations:** 1 Institute of Medical Microbiology, Immunology and Parasitology (IMMIP), University Hospital Bonn, Sigmund-Freud-Str. 25, D-53127 Bonn, Germany; 2 Department of Radiology, University Hospital Bonn, Sigmund-Freud-Str. 25, D-53113 Bonn, Germany; 3 Department of Neurosurgery, University Hospital Bonn, Sigmund-Freud-Str. 25, D-53113 Bonn, Germany; 4 Institute of Neuropathology, University Hospital Bonn, Sigmund-Freud-Str. 25, D-53113 Bonn, Germany

**Keywords:** *Fusobacterium nucleatum*, Brain abscess, Brain death, Amphetamine abuse, 16s rDNA PCR

## Abstract

**Background:**

*Fusobacterium nucleatum* is a strict anaerobic microorganism that causes disease entities such as periodontal and soft tissue abscesses, pulmonary and intraabdominal infections and very rarely intracerebral infections.

**Case presentation:**

Here, we report the rare case of a previously healthy 25-year-old German man with a cerebellar abscess caused by *Fusobacterium nucleatum* that resulted in rapid brain death. Toxicological screening showed positivity for amphetamines and cannabis. The diagnosis was obtained by polymerase chain reaction amplification of bacterial deoxyribonucleic acid in cerebrospinal fluid.

**Conclusions:**

In drug users clinicians should think about rare causes of brain abscesses/meningitis. Early diagnosis is necessary and justifies the use of molecular techniques.

## Background

*Fusobacterium nucleatum* is a strictly anaerobic microorganism that is indigenous to the human oral cavity, where it coexists with more than 500 other species [1]. It can also be found in the gastrointestinal, urogenital and upper respiratory tracts. It belongs to the family of *Bacteroidaceae*. Fusobacteria have a proteolytic metabolism with only low-level glucose acid-formation. Cells are gram negative rod-shaped with pointed ends.

*Fusobacterium nucleatum* has been reported to play a causative role in infections such as sinusitis, periodontal disease, pulmonary infections, intraabdominal infections, i.e. appendicitis and liver abscesses, and it was detected in a psoas abscess, septic arthritis, osteomyelitis and endocarditis [2-6]. Severe clinical courses, predominantly in adolescents, have been described. Clinical syndromes caused by *Fusobacterium* spp. are collectively referred to as necrobacillosis [7]. The literature documents that brain affection is, however, extremely rare [8-14].

## Case presentation

Here we report the case of a 25-year-old German man, with no prior history of chronic illness or substance abuse. Nevertheless, toxicological screening was positive for cannabis and amphetamines. After a 72 h period of progressively increasing headache and psychiatric alteration he was admitted to a community hospital in a delirant state with somnolent phases. The patient was in good physical condition with aggressive fits, co-operative and orientated when awake and conscious and showed no signs of acute infection apart from an elevated C-reactive protein (61.6 mg/l) and leukocytes (21.6 G/l). Additional signs as fever, tachycardia or tachypnoe to diagnose severe inflammatory response syndrome (SIRS) were not seen. Upon physical examination there were no signs of meningism or impairment of motor or sensory function. Cranial nerve examination was discreet, pupils reactive to light and isocore.

**Figure 1:**
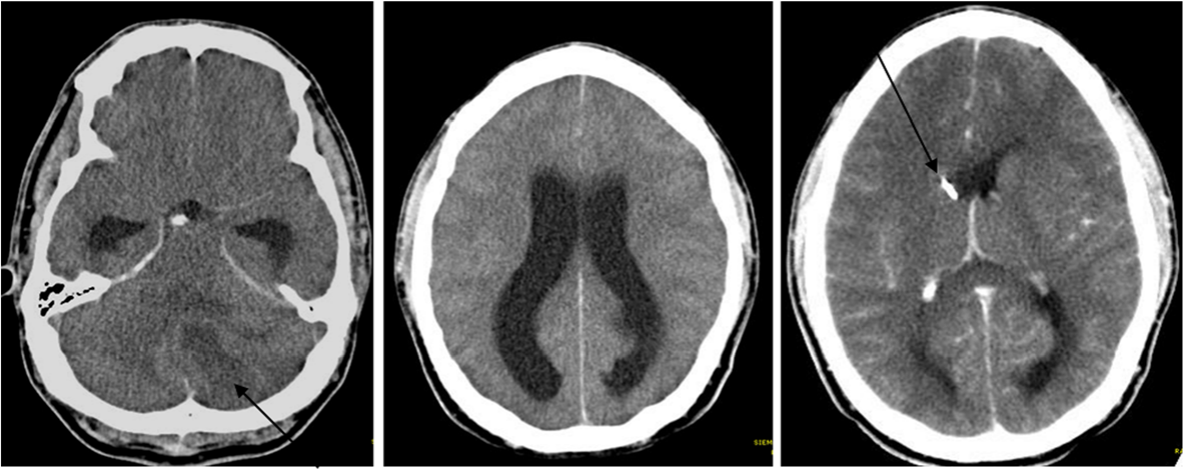
**Computer tomography (CT) scan of the brain.** CT scan of the cerebrum (left). White arrow indicates space occupying lesion. Cerebrum with widening of the side ventricles compatible with a hydrocephalus (middle) of the cerebrum after placement of an extraventricular drainage (EVD) (right). Black arrow indicates EVD in the side ventricle.

Clinical examination showed multiple scratches and infected pustules on both outer upper legs and arms. According to the family this situation had been longstanding and was not acute. The dermatologists classified these lesions as *Prurigo subacuta.* Six h after hospitalization the patient deteriorated rapidly to Glascow coma scale (GCS) 3, was intubated and put on mechanical ventilation. Pupils were dilated. A native computer tomography (CT) brain scan showed massive brain swelling with brain stem compression and a space occupying lesion (SOL) in the left cerebella hemisphere causing an occlusive hydrocephalus (Figure 1).

An external ventricular drainage (EVD) was inserted immediately and produced cerebrospinal fluid (CSF) under very high pressure. Cell counts and laboratory analysis of CSF showed increased polymorphic granulocytes (1116/mm^3^), lymphocytes (115/mm^3^) and red blood cells (3000/mm^3^). The lactate and protein concentration was markedly elevated (17 mmol/l and 12967.1 mg/dl, respectively) and glucose levels were low (<1 mg/dl).

**Figure 2:**
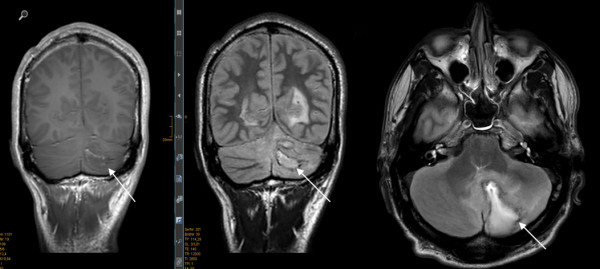
**Magnetic resonance imaging (MRI) scan of the brain.** T1 images with contrast medium (left and middle) and T2 images (right). White arrows indicate cerebellar abscess formation.

Despite these findings antibiotic therapy with flucloxacillin, ceftazidime and metronidazole had no clinical effect. Microscopy of gram stained CSF specimen showed a massive number of leucocytes but no bacteria. Aerobic and anaerobic cultures were performed but remained negative. Specific polymerase chain reactions (PCR) excluded typical pathogens causing meningitis, e.g. *Neisseria meningitides* and *Streptococcus pneumoniae*[15,16]. However, a PCR product was detected with 16S ribosomal deoxyribonucleic acid (rDNA) primers as previously described in [17]. PCR product sequencing detected genomic deoxyribonucleic acid (DNA) from *Fusobacterium nucleatum* with 98% homology.After the positioning of an EVD a CT brain scan with contrast agent was performed revealing no enhancement of the SOL but suspected occlusion of the basilar artery. The digital subtraction angiography (DSA) performed afterwards showed absence of perfusion compatible with brain death. Subsequently, an magnetic resonance imaging (MRI) was performed to complete the diagnostic imaging. Figure 2 documents abscess formation in the left cerebellum hemisphere. Despite maximal therapeutical effort to lower the intracerebral pressure reactivity of pupils could not be restored and brain stem reflexes vanished within hours. Brain death was declared within 12 hours after the initial drop in GCS.

Pathological examination revealed an abscess in the left cerebella hemisphere with cerebellar oedema and brain stem herniation. There were signs of embolism of both main trunks of the pulmonary arteries. Microscopic analysis of the heart muscle showed hypereosinophilia as seen in necrosis and apoptosis.

## Discussion

In the majority of cases aerobic pathogens are thought to be responsible for meningitis or brain abscesses. Nevertheless, over the last decades an increase in the detection of anaerobic pathogens causing meningitis has been observed. It is, however, unclear whether this is due to better processing of the specimen and increased sensitivity of detection methods. In the literature there are many reports about meningitis caused by anaerobes, but only about 30 reports on meningitis caused by *Fusobacterium* spp.. Overall, the incidence of brain infections caused by strictly anaerobic bacteria may have been underestimated in the past, and this neglect might account for delays in diagnosis or failure to identify the causative pathogen. Albeit several reports about brain abscesses caused by *Fusobacterium nucleatum* report successful recovery under conservative management, delay of effective antibiotic treatment can increase the severity of disease and lethality [10,13,18].

Notably, one difficulty in our case was that the patient showed no typical signs for meningitis. Pathological examination confirmed this revealing only few immune cells infiltrating the meninges. Moreover, the patient showed symptoms that were well-compatible with drug intoxication. This might have contributed to the delay in the performance of the CT scan and the diagnosis. Next to the increased risk for abscess formation associated with intravenous drug abuse drug-induced immune suppression (described for both amphetamine and cannaboids) might predispose for infections with unusual clinical course and symptoms [19,20]. Moreover, skin eruptions classified as *Prurigo subacuta* could originate from amphetamine abuse [21]. Radiological examination showed yet no classical enhancement pattern of contrast agent in the abscess border but a rather atypical abscess formation, thus making the differential diagnosis between abscess, tumor or hematoma difficult.

Previous studies report that only 6% of brain abscesses caused by bacteria arise from infection with *Fusobacterium* spp. [11]. A review of the literature showed that there are only few descriptions of *Fusobacterium nucleatum* brain abscesses with only seven papers and a total number of 16 patients documented in the MEDLINE. Table 1 summarizes the available reports about meningitis caused by *Fusobacterium nucleatum*. In contrast to the case presented here all other patients survived. The majority of patients received both surgery and antibiotic treatment. An interesting observation is that 87.5% of the patients reported were of male gender, but the reasons remain obscure. Diagnosis of *Fusobacterium nucleatum* infection in most cases was based on culture (81.25%) and in only 18.75% on nucleic acid amplification techniques (NAT) [9-14,18]. Fourteen out of 16 culture positive specimens were derived from tissue samples or pus, which shows that an optimal culture result depends on the provision of an optimal specimen, such as tissue probes or pus obtained with invasive methods.

**Table 1:** **Summary of published reports on meningitis and brain abscesses caused by *Fusobacterium nucleatum***

	**Age/sex**	**Surgical treatment**	**Antibiotic therapy**	**Neurological symptoms**	**Detection of *F. nucleatum***	**Therapeutic outcome**
Stefániková L *et al.* 2008 [18]	41/M	NO	cefotaxime, metronidazole, subsequently co-trimoxazole and rifampin	headache, fever, slightly hemiparesis of the right leg	PCR	survived, total recovery
Kai A *et al*. 2008 [12]	47/M	NO	ceftriaxone, metronidazole	headache, neck pain, fever, vomiting, transient loss of consciousness	culture	survived, total recovery
Taguchi Y *et al*. 1981 [14]	28/M	YES	penicillin G, chloramphenicol	pain left temporal region, general convulsion	culture	survived, total recovery
Heckmann JG *et al.* 2003 [10]	59/F	NO	ceftriaxone, metronidazole	confusion, neck stiffness, history of moderate ethanol	PCR	survived, total recovery
Keller PM *et al.* 2010 [13]	29/M	YES	ceftriaxone, metronidazole	seizure, frontal headache	PCR	survived, residual chronic headache
Hsieh MJ *et al.* 2007 [11]	71/F	YES	penicillin G, chloramphenicol	headache, fever, seizure, visual disturbance, hemiparesis	culture	survived, hemiparesis
	58/M	YES	penicillin G, metronidazole	headache, fever, visual disturbance, stiff neck	culture	survived, hemiparesis, homonymous hemianopia
	64/M	YES	penicillin G, chloramphenicol	fever, seizure	culture	survived, total recovery
	34/M	YES	ceftriaxone, metronidazole	headache, fever, vomiting, stiff neck	culture	survived, total recovery
	52/M	YES	ceftriaxone, metronidazole	hemiparesis	culture	survived, wheelchair-bound
	51/M	YES	ceftriaxone, vancomycin, metronidazole	fever, consciousness disturbance	culture	survived, total recovery
Han XY *et al.* 2003 [9]	45/M	YES	penicillin G, metronidazole, ceftazidime	headache, short term memory loss, slurred speach	culture	survived
	56/M	YES	vancomycin, ceftazidime, metronidazole	headache, confusion, disorientation, left visual field deficits	culture	survived
	74/M	YES	vancomycin, metronidazole, cefepime	garbled speech, incomplete paresis of the right hand	culture	survived
	48/M	YES	ceftriaxone, metronidazole	headache, speech difficulty, confusion, memory loss	culture	survived
	59/M	YES	vancomycin, ceftazidime, metronidazole	memory loss, right-side weakness, aphasia	culture	survived

*Abbreviations*: M (male), F (female), PCR (polymerase chain reaction).

As PCR is not performed routinely in many laboratories it is likely that a certain percentage of cases remain undetected. PCR-mediated amplification and sequencing of 16S ribosomal DNA might be of special usefulness in cases that remain negative in culture, possible due to prior antibiotic treatment or only low bacterial load such as that in the CSF of our patient. Notably, a biopsy of the brain abscess was not available during the short clinical course. As evidenced by the case presented in the absence of a suitable biopsy the use of NAT can enforce the diagnosis.

Until today many acute cases of inflammatory brain pathologies lead to death very rapidly. This case and the review of the literature show that - albeit rare - anaerobic microorganisms should not be disregarded in the differential diagnosis of intracranial infection and inflammation. Antibiotic therapy in brain inflammation should, therefore, cover anaerobic microorganisms. Drug abuse, including amphetamines and cannabinoids, predisposes for infections by suppressing immune function. Drug intoxication additionally obscures clinical symptoms relevant for diagnosis.

## Conclusion

The widespread use of narcotic substances should, therefore, make us suspect of brain abscesses with rare pathogens in patients with unusual neurological symptoms. Severe cases, such as the one described in this study, justify early invasive procedures to obtain adequate specimen and rapid molecular approaches to obtain the diagnosis and initiate adequate treatment. Clinicians should stay in close contact with microbiologists to receive rapid results and therefore to launch sufficient therapy.

## Consent

Written informed consent was obtained from the patient’s father for publication of this Case Report and any accompanying images. A copy of the written consent is available for review by the Editor-in-Chief of this journal.

## Competing interests

The authors declare that they have no competing interests.

## Authors’ contributions

GH and IBD wrote the manuscript. KG was involved in PCR diagnostic, VK and AB have been involved in clinical treatment of the patient and KK has been involved in post mortem pathological examination. KG, VK, AB and KK have been involved in critically revising the manuscript. All authors read and approved the final manuscript.
